# Combined Toxicity of Ofloxacin and Sulfamethoxazole at Environmentally Relevant Concentrations in Mosquitofish: Histopathological Damage, Oxidative Stress, and Gut Microbiota Alterations

**DOI:** 10.3390/toxics14060457

**Published:** 2026-05-23

**Authors:** Xu Ding, Xin Li, Haojie Liu, Zhong Li, Yangchun Xia, Yanpeng Liang, Honghu Zeng, Xiaohong Song

**Affiliations:** 1 Guangxi Key Laboratory of Environmental Pollution Control Theory and Technology, Guilin University of Technology, Guilin 541006, China; 17390495924@163.com (X.D.);; 2 University Engineering Research Center of Watershed Protection and Green Development, Guilin University of Technology, Guilin 541006, China; 3 Key Laboratory of Carbon Emission and Pollutant Collaborative Control, Education Department of Guangxi Zhuang Autonomous Region, Guilin University of Technology, Guilin 541006, China

**Keywords:** ofloxacin, sulfamethoxazole, histopathology, oxidative stress, gut microbiota

## Abstract

Ofloxacin (OFL) and sulfamethoxazole (SMX) are common co-occurring antibiotic contaminants in aquatic environments, yet their long-term combined toxicity to freshwater fish remains poorly elucidated. In this study, adult mosquitofish (*Gambusia affinis*) were used as a model to investigate histopathological alterations, oxidative stress responses, gene expression, and gut microbiota changes after 30 days of exposure to environmentally relevant concentrations of OFL and SMX (0 ng/L, 50 ng/L, 1 μg/L, and 20 μg/L), either individually or in combination. The results showed that both single and combined exposures induced liver and intestinal damage. Oxidative stress responses exhibited clear tissue specificity, with activation of antioxidant defenses in the liver, whereas the intestine was mainly characterized by decreased SOD and GST activities, as well as reduced MDA content. Changes in gene expression were relatively limited, with significant alterations observed only in hepatic *sod2* and *hsp90* and intestinal *hsp70* in certain treatment groups. Gut microbiota analysis showed that OFL exerted a stronger disruptive effect than SMX, as reflected by increased alpha diversity, reduced abundance of core genera, and functional remodeling, whereas combined exposure triggered weaker microbial community restructuring relative to single exposures. Overall, OFL and SMX induced tissue-specific toxicity in mosquitofish by causing tissue injury, oxidative stress imbalance, and gut microbiota dysbiosis, with OFL showing the stronger overall effect.

## 1. Introduction

In recent years, the widespread occurrence of antibiotic residues in aquatic environments and their associated ecological risks have emerged as a pressing global concern [1]. Previous studies have confirmed that antibiotics can induce oxidative stress, trigger immune and inflammatory responses [2], and disrupt the ecological and metabolic functions of intestinal microbiota in various aquatic organisms [3]. Ofloxacin (OFL) and sulfamethoxazole (SMX), typical representatives of fluoroquinolone and sulfonamide antibiotics, respectively [4], are commonly detected in aquatic environments worldwide at concentrations ranging from ng/L to μg/L [5,6]. This widespread contamination is primarily attributed to their extensive use in medical treatment and livestock breeding, together with their environmental persistence.

These two antibiotics frequently coexist as mixed contaminants in aquatic systems, and their synergistic or antagonistic interactions may pose long-term threats to aquatic organisms and ecosystem stability, with risks further amplified through food chain biomagnification [7]. Empirical monitoring data highlight the severity of this issue: among 1052 river sampling sites worldwide, SMX concentrations exceeded the predicted no-effect concentration (PNEC) for aquatic organisms at 140 sites, accounting for 13% of the total sites [8]. In the Pearl River Delta aquaculture region of China, sulfamethazine residues as high as 26.2 μg/kg have been detected in fish muscle tissues [9], while ofloxacin was measured at a concentration of 31.9 ng/L in urban groundwater in Wuhan, China [10]. A large proportion of antibiotics used in human and veterinary medicine eventually enter natural environments, exerting adverse effects on multiple components of aquatic ecosystems, including freshwater algae, planktonic microbes, macrophytes, zooplankton, and fish [1].

Antibiotics can induce transcriptional and enzymatic alterations as well as mitochondrial damage in fish cells, and such physiological disturbances further lead to abnormal histological changes and even mortality in fish [11]. A growing body of evidence has confirmed that antibiotics exert substantial detrimental effects on aquatic organisms by disrupting key life processes, including development, growth and reproduction [12], and by impairing physiological and biochemical pathways and immune function [13]. For example, exposure of *Caenorhabditis elegans* to 2.6 ng/L ofloxacin induced reproductive toxicity, characterized by multi-generational oscillatory effects and lingering transgenerational impacts [14]. In fish species, norfloxacin exposure altered the activities of EROD, GST and SOD enzymes in male goldfish (*Carassius auratus*) and zebrafish *(Danio rerio*), indicating the activation of the antioxidant defense system [9]. However, excessive antibiotic exposure surpasses adaptive regulatory thresholds, impairing the growth performance of aquatic organisms and even causing fish poisoning, developmental deformities and other pathological disorders or diseases [15].

Further targeted studies on specific antibiotics and fish models have revealed more nuanced toxic effects. Excessive doses of sulfamethoxazole triggered kidney injury in grass carp (*Ctenopharyngodon idella*), accompanied by inflammation and pyroptosis [16]. Similarly, exposure to 260 ng/L sulfamethoxazole induced oxidative stress, suppressed innate immunity, activated inflammatory and detoxification responses, reduced antioxidant capacity, and triggered lipid peroxidation in the intestinal and hepatic tissues of Nile tilapia (*Oreochromis niloticus*); this exposure also upregulated the mRNA expression levels of the *sod* gene in these tissues [17]. Beyond visceral and peripheral tissues, certain antibiotics can penetrate the blood–brain barrier and induce cerebral oxidative stress, resulting in neuronal damage and dysfunction [2].

The intestine, a pivotal organ responsible for digestion and nutrient absorption, as well as a primary route of toxin exposure [18], is highly susceptible to antibiotic-induced damage. Antibiotic exposure can disrupt the composition and structure of the intestinal microbiota in zebrafish, further inducing behavioral abnormalities, including hyperactivity, anxiety, and depressive-like phenotypes [19]. In zebrafish exposed to mixed antibiotic formulations, growth retardation is closely associated with colorectal dysfunction, intestinal oxidative stress, and cellular apoptosis [20]. Moreover, antibiotic-induced redox homeostasis imbalance and abnormal intestinal permeability are significantly correlated with reduced survival rates in Nile tilapia [17].

Notably, the toxic effects of antibiotics on organisms often differ substantially between individual and combined exposure scenarios [21]. The cumulative effects of antibiotic mixtures may be antagonistic, additive, or synergistic [6]. For instance, zebrafish larvae exposed to environmentally relevant concentrations (0, 1, and 10 μg/L) of fluoroquinolone and sulfonamide antibiotics for 24 h exhibited distinct neurobehavioral and oxidative stress responses under single and mixed treatments, with antibiotic mixtures exacerbating toxic effects compared with individual compounds [22]. In contrast, combined exposure to fluoroquinolones (FQs) and tetracyclines (TCs) in zebrafish embryos and larvae produced toxicological effects comparable to, or slightly less pronounced than, those induced by TCs alone; notably, TCs acted as the dominant component in the mixture, and their co-occurrence with FQs primarily resulted in antagonistic interactions [23]. Despite these findings, current knowledge of antibiotic-induced tissue-level effects in vertebrates remains limited, especially for long-term exposure to antibiotic mixtures at environmentally relevant concentrations and their effects on wild fish species.

To address this research gap and further explore the toxic mechanisms of individual and combined antibiotics, the mosquitofish *Gambusia affinis*, a small freshwater fish species, was selected as the test organism. The mosquitofish is widely utilized in ecotoxicological research due to its broad distribution, rapid reproduction, and high sensitivity to pollutants [24]; thus, this species is well-suited for investigating antibiotic co-contamination effects. Specifically, this study aimed to investigate the individual and combined effects of environmentally relevant concentrations of ofloxacin (OFL) and sulfamethoxazole (SMX) (0 ng/L, 50 ng/L, 1 μg/L, 20 μg/L) on tissue histopathology, intestinal microbiota, antioxidant biomarkers, and gene transcription in adult mosquitofish following 30 days of continuous exposure. By integrating multidimensional indicators spanning physiology, molecular biology, and microbiome analysis, this research elucidates the toxicity pathways of individual versus combined antibiotic exposure in mosquitofish. The findings could provide scientific evidence for assessing aquatic ecological risks of antibiotic co-contamination while establishing a theoretical foundation for defining antibiotic safety thresholds.

## 2. Materials and Methods

### 2.1. Chemicals

Ofloxacin (purity > 98%) and sulfamethoxazole (purity > 98%) were purchased from TCI Chemical Industry Development Co., Ltd. (Shanghai, China). Ethylenediaminetetraacetic acid disodium salt (EDTA-2Na), hydrochloric acid (HCl), and sodium hydroxide (NaOH) (all of analytical grade), as well as formaldehyde solution and dimethyl sulfoxide (DMSO) (purity ≥ 99.5%), were obtained from Xilong Scientific Co., Ltd. (Shantou, China). Methanol (purity > 99.9%, HPLC grade) was supplied by Merck KGaA (Darmstadt, Germany). Formic acid (purity > 98.0%, HPLC grade) and ammonia solution (HPLC grade) were purchased from Aladdin Reagent Co., Ltd. (Shanghai, China).

### 2.2. Test Organisms

The mosquitofish used in this study were approved by the Animal Care and Use Ethics Committee of the Guilin University of Technology, and all experimental operations were strictly conducted in accordance with the relevant management specifications for animal experiments. A total of 500 mosquitofish were purchased from a local aquaculture farm in Guilin and acclimated and reared in a laboratory aquatic organism rearing system for two weeks. The rearing environment was fully controllable: the water temperature was stably maintained at 24 ± 2 °C, the photoperiod was 14 h:10 h (light:dark), brine shrimp were fed twice a day at regular intervals, and dead individuals, residual feed, and metabolic excreta were promptly fished out and cleaned up. During acclimatization and rearing, a total of 11 fish died, with mortality below 3%. After the fish had fully adapted to the laboratory environment, 240 healthy, active adult mosquitofish with consistent morphology and size were selected for the formal experiment. The average body mass of the experimental fish was (0.16 ± 0.05) g, and the average body length was (2.23 ± 0.19) cm.

### 2.3. Exposure Test and Sample Collection

Ofloxacin (OFL) and sulfamethoxazole (SMX) have been widely reported in various water bodies (e.g., surface waters and wastewaters) across regions, with residue concentrations generally ranging from ng/L to μg/L levels [1]. Specifically, OFL residues have been detected at up to 68 μg/L in hospital effluent water [25] and 11.7 μg/L in river water [26], while SMX residues have been recorded at up to 12.8 μg/L in effluent water [27] and 11.92 μg/L in river water [28].

For the experiment, OFL and SMX were prepared as stock solutions in dimethyl sulfoxide (DMSO), stored at 4 °C in the dark. The final concentration of DMSO in all exposure solutions was maintained at 0.004% (*v*/*v*) with a mass fraction < 0.1%. Ten experimental groups were established, including three OFL-only exposure groups (50 ng/L, 1 μg/L, 20 μg/L), three SMX-only exposure groups (50 ng/L, 1 μg/L, 20 μg/L), three equimolar OFL + SMX co-exposure groups (50 ng/L OFL + 50 ng/L SMX, 1 μg/L OFL + 1 μg/L SMX, 20 μg/L OFL + 20 μg/L SMX), and a DMSO control group (CK). Each group included three biological replicates, with eight adult female mosquitofish per replicate.

All experimental water was tap water aerated for more than 2 days, with a pH of 7.1–7.4, water temperature of (24 ± 2) °C, and dissolved oxygen ≥ 5.0 mg/L. A semi-static water change method was adopted, with 1/2 of the experimental solution replaced daily. No fish mortality was observed throughout the 30-day exposure period. Subsequently, fish were sampled and dissected on ice. The liver and intestine tissues of mosquitofish were quickly isolated under a stereomicroscope. Specifically, 2 fish per beaker were fixed in 10% formaldehyde fixative for subsequent H&E histopathological sectioning; 3 fish per beaker were snap-frozen in liquid nitrogen and stored at −80 °C for enzyme activity assays; an additional 3 fish were immersed in RNA preservation solution (Takara) and stored at −20 °C for total RNA extraction. Additionally, intestinal contents were collected from the 20 μg/L OFL group, 20 μg/L SMX group, 20 μg/L OFL + 20 μg/L SMX co-exposure group, and CK group. These samples were flash-frozen in liquid nitrogen and stored at −80 °C for subsequent analysis.

### 2.4. Detection of Antibiotic Concentrations in Water Samples

During the 30-day exposure experiment, water samples were randomly collected every 10 days, resulting in a total of three sampling events. Following collection, the samples were filtered through 0.45 μm glass fiber filters. The concentrations of OFL and SMX were determined using ultra-high-performance liquid chromatography coupled with triple quadrupole tandem mass spectrometry (UHPLC-QqQ-MS/MS, Waters Corp., Milford, MA, USA). The limits of detection (LOD) and quantification (LOQ) for both OFL and SMX were 1 ng/L and 3 ng/L, respectively. To ensure the accuracy and reliability of the results, quality control measures were implemented, including method spike blanks, method blanks, matrix blanks, and matrix spike experiments.

### 2.5. Histopathological Section

The dissected liver and intestinal tissues of the mosquitofish were embedded in optimal cutting temperature (OCT) compound. The embedded tissues were sectioned at a thickness of 6 μm using a LEICA CM1850 cryostat (Leica Biosystems, Wetzlar, Germany). Subsequent to sectioning, the tissue sections were stained with hematoxylin and eosin (H&E), mounted with neutral balsam, and then observed and photographed under an automatic digital slide scanner (Gscan-20, G cell technology, Guangzhou, China). Subsequently, histopathological semi-quantitative evaluation was performed on the liver and intestinal tissues.

### 2.6. Detection of Antioxidant Enzyme Activity

Ice-cold physiological saline was added to the tissues at a ratio of 1:9 (*w*/*v*, tissue weight: saline volume). The mixture was homogenized using a cryogenic tissue grinder (Shanghai Jingxin Instrument Co., Ltd., Shanghai, China) under low-temperature conditions. The homogenate was centrifuged at 4 °C and 2500 rpm for 15 min, and the supernatant was collected and diluted to a 1% tissue homogenate. Subsequent determinations of superoxide dismutase (SOD), catalase (CAT), and glutathione S-transferase (GST) activities, as well as malondialdehyde (MDA) and total protein (TP) contents, were performed using commercial assay kits from Nanjing Jiancheng Bioengineering Institute (Nanjing, China), with specific operations carried out in accordance with the manufacturer’s instructions.

### 2.7. mRNA Expression of Antioxidant-Related Genes

Total RNA was extracted from the liver and intestine tissues of adult mosquitofish using the RNAiso™ Plus reagent (TaKaRa Bio Inc., Kusatsu, Shiga, Japan). RNA quality and concentration were assessed using a Quawell Q5000 ultra-micro-spectrophotometer (Quawell Technology Inc., San Jose, CA, USA). Genomic DNA was eliminated, and cDNA was synthesized from total RNA using a PrimeScript™ RT Reagent Kit with gDNA Eraser (TaKaRa Bio Inc., Kusatsu, Shiga, Japan). Quantitative real-time PCR (qPCR) was performed on a QuantStudio3 system (Thermo Fisher Scientific, Waltham, MA, USA) with SYBR™ Premix Ex Taq™ II (Tli RNaseH Plus) (TaKaRa Bio Inc., Kusatsu, Shiga, Japan). The qPCR primer sequences are shown in Table S1. The 2^−ΔΔCT^ method [29] was used to analyze the relative mRNA expression levels of antioxidant-related genes (*gst*, *hsp70*, *hsp90*, *sod2*, and *cat*) and the reference gene *gapdh* in the liver and intestinal tissues.

### 2.8. 16S rRNA Sequencing and Analysis of Intestinal Microbiota

Total genomic DNA was extracted from fish intestinal contents using a HiPure Stool DNA Kit (Guangzhou Meiji Biotechnology Co., Ltd., Guangzhou, China). DNA quality and purity were evaluated using a NanoDrop spectrophotometer (NanoDrop 2000, Thermo Fisher Scientific, Waltham, MA, USA), while nucleic acid integrity was verified via 1% agarose gel electrophoresis (Bio-Rad Laboratories, Inc., Hercules, CA, USA).

The conserved regions of bacterial rDNA were amplified using specific primers tagged with barcodes. PCR amplification products were recovered via gel excision and quantified using a QuantiFluor™ Fluorometer (Promega Corporation, Madison, WI, USA). Purified amplicons were mixed in equal proportions, ligated with sequencing adapters to construct the sequencing library, and then subjected to high-throughput sequencing on the Illumina PE250 platform.

Raw reads were filtered, assembled into tags, and refined to generate clean tags; following clustering and chimera removal, effective tags were used for OTU clustering and abundance quantification. For microbial community analysis, alpha diversity was analyzed using QIIME (version 1.9.1). Venn diagrams, principal coordinate analysis (PCoA), redundancy analysis (RDA), UPGMA clustering, bacterial indicator analysis, and heatmap construction were performed in R (version 4.2). KEGG metabolic pathway prediction was conducted using PICRUSt (version 2.1.4).

### 2.9. Data Analysis

Data are presented as mean ± standard error of the mean (Mean ± SEM) and processed using SPSS Statistics 22 software. One-way analysis of variance (ANOVA) combined with Duncan’s multiple range test was performed for statistical comparisons, and data visualization was generated using GraphPad Prism 9. In the figures, distinct lowercase letters (a, b, c, d, e) represent significant differences between groups (*p* < 0.05), and samples without letter labeling indicate no significant difference compared with the control group.

## 3. Results

### 3.1. Actual Antibiotic Concentrations in Exposure Water

Nominal and measured concentrations of OFL and SMX in all exposure solutions are summarized in Table S2. The measured concentrations in both single and combined treatments were generally consistent with the nominal values, with relative deviations within an acceptable range for OFL and SMX of 2.30–16.91% and 4.39–43.80%, respectively. Noticeable degradation was observed in the 1 μg/L SMX treatment, with deviations of 35.8% in the single-exposure group and 43.80% in the mixed-exposure group. However, because the three exposure levels were established using a 20-fold concentration gradient (50 ng/L, 1 μg/L, and 20 μg/L), the treatments still clearly represented environmentally relevant ng/L- to μg/L-level exposure. Accordingly, all subsequent analyses of biological effects were conducted based on the nominal exposure concentrations.

### 3.2. Histopathological Lesions in Different Tissues

#### 3.2.1. Hepatic Histopathology

**Figure 1:**
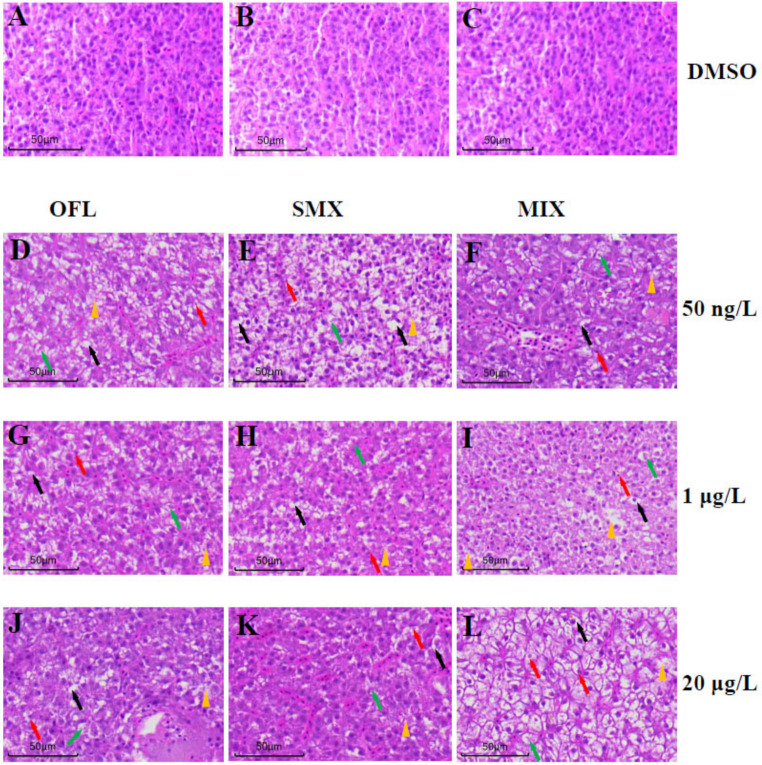
Hepatic histological sections of mosquitofish after 30 days of exposure in the following groups: DMSO control group (**A**–**C**); OFL single-exposure groups at concentrations of 50 ng/L (**D**), 1 μg/L (**G**), and 20 μg/L (**J**); SMX single-exposure groups at 50 ng/L (**E**), 1 μg/L (**H**), and 20 μg/L (**K**); and combined-exposure groups (OFL + SMX) at 50 ng/L (**F**), 1 μg/L (**I**), and 20 μg/L (**L**). Black arrows: cytoplasmic vacuolation of hepatocytes; orange arrowheads: cytolysis; red arrows: condensed nuclei; green arrows: nuclear fragmentation. Scale bar: 50.0 μm.

Histological analysis of hepatic tissues revealed distinct structural damage in all antibiotic-treated groups compared to the control group. In the control group (Figure 1A–C), hepatocytes were regularly arranged with intact cell boundaries, round nuclei, and homogeneous cytoplasm with occasional morphological changes.

In the OFL-treated groups (Figure 1D,G,J), injured hepatocytes exhibited cytolysis, cytoplasmic vacuolation, condensed nuclei, nuclear migration and disordered arrangement. According to the semi-quantitative evaluation (Table S3), hepatic damage was aggravated in a concentration-dependent manner from 50 ng/L to 20 μg/L.

In the SMX-treated groups (Figure 1E,H,K), injuries included cytolysis, cytoplasmic vacuolation, and pyknosis. Lesions were mild at all concentrations (Table S3) with no obvious concentration-dependent trend, although the 50 ng/L SMX group showed more serious vacuolation.

In the OFL + SMX mixture groups (Figure 1F,I,L), injuries were more severe than in the single-exposure groups, especially the SMX groups. Damage gradually intensified with increasing concentration (Table S3), and the 20 μg/L mixture caused the most marked structural damage, accompanied by extensive cytolysis and cytoplasmic vacuolation.

Collectively, OFL caused more severe hepatic damage than SMX in a concentration-dependent manner (Table S3). Combined exposure to OFL and SMX, especially at the 20 μg/L group, induced more serious hepatic injury than either antibiotic alone.

#### 3.2.2. Intestinal Histopathology

**Figure 2:**
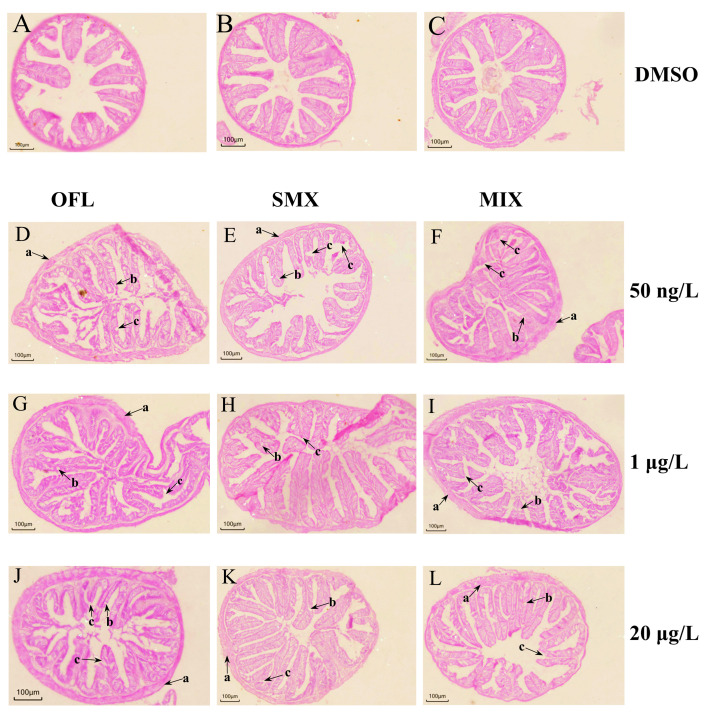
Intestinal histological sections of mosquitofish after 30 days of exposure in the following groups: the DMSO control group (**A**–**C**); OFL single-exposure groups at concentrations of 50 ng/L (**D**), 1 μg/L (**G**), and 20 μg/L (**J**); SMX single-exposure groups at 50 ng/L (**E**), 1 μg/L (**H**), and 20 μg/L (**K**); and combined-exposure groups (OFL + SMX) at 50 ng/L (**F**), 1 μg/L (**I**), and 20 μg/L (**L**). Pathological alterations included thickening of the muscular layer (a), vacuolation (b), and villus rupture (c). Scale bar: 100 μm.

The intestinal tissues of the mosquitofish in the control group showed an intact morphological structure: well-organized intestinal folds with clear boundaries, continuous and intact mucosal epithelium, and a uniformly thick muscular layer (Figure 2A–C). In contrast, all antibiotic exposure groups exhibited varying degrees of intestinal structural damage and appeared more fragile (Figure 2D–L). The main pathological alterations included thickening of the intestinal muscular layer, cellular vacuolation, and villous rupture. Lesions were mild across all concentrations and exposure regimens, with no obvious concentration-dependent trend (Table S4).

### 3.3. Antioxidant Enzyme Activities in Different Tissues

**Figure 3:**
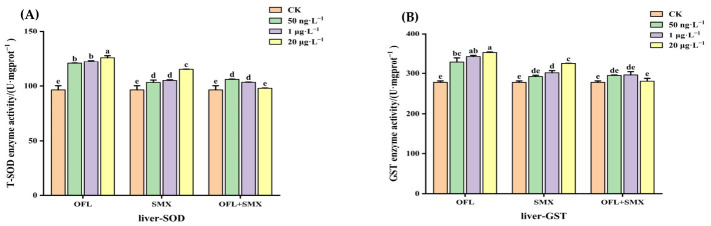

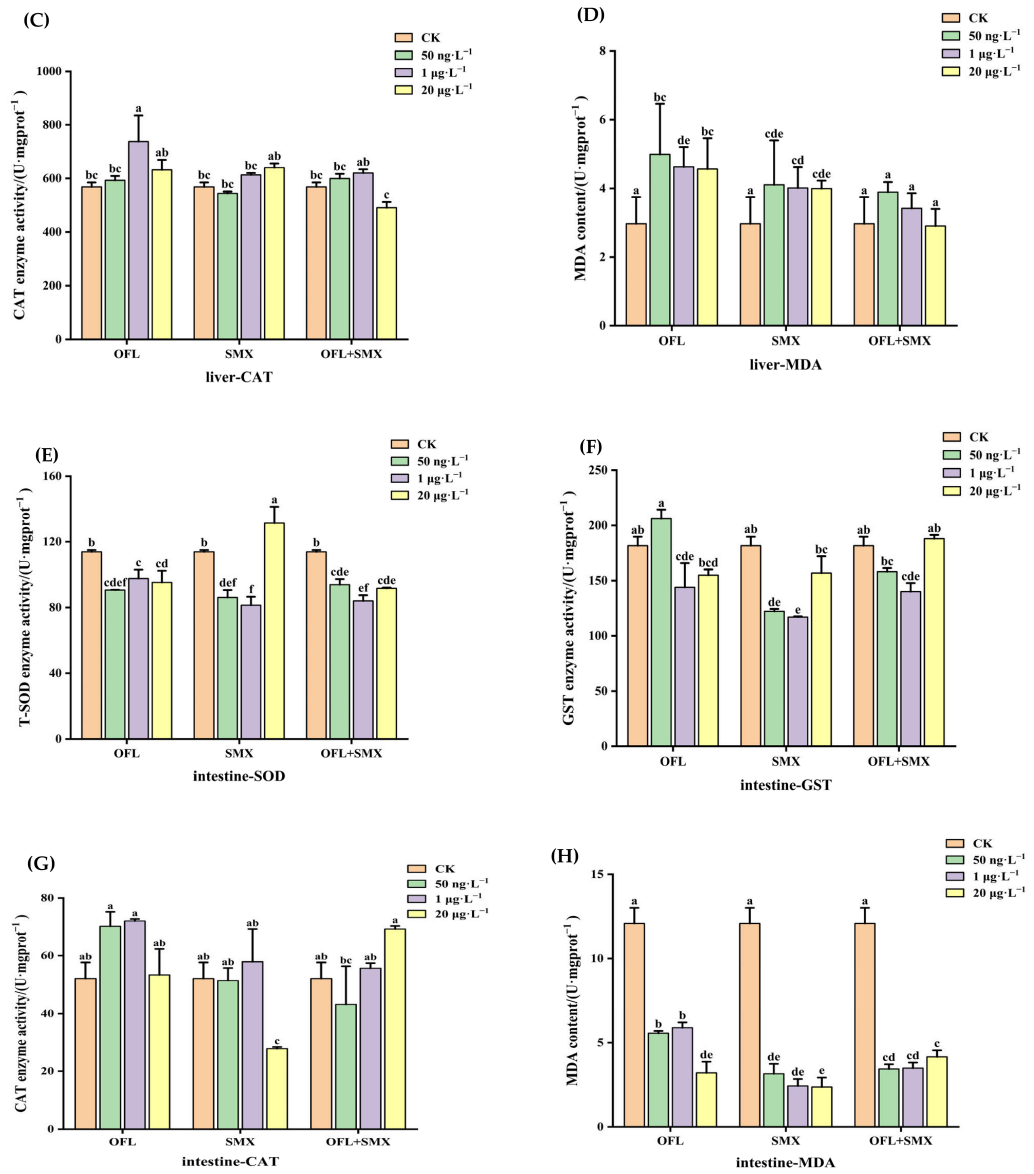
Effects of ofloxacin and sulfamethoxazole alone and in combination on antioxidant indices in the liver and intestine of mosquitofish. The liver: (**A**) superoxide dismutase (SOD) activity, (**B**) glutathione S-transferase (GST) activity, (**C**) catalase (CAT) activity, (**D**) malondialdehyde (MDA) content. The intestine: (**E**) SOD activity, (**F**) GST activity, (**G**) CAT activity, (**H**) MDA content. Dfferent lowercase letters (a, b, c, d, e) indicate significant differences among groups (*p* < 0.05).

Antioxidant enzyme activities (SOD, GST, CAT) and MDA content in the liver and intestine tissues showed tissue-specific and concentration-dependent responses to OFL and SMX single and combined exposures, with significant antibiotic interaction effects (Figure 3).

In the liver, SOD activity (Figure 3A) was significantly upregulated in all OFL and SMX single-exposure groups (*p* < 0.01), while in the combined-exposure groups, only the low (50 ng/L) and medium (1 μg/L) concentrations induced a significant increase (*p* < 0.01), and the high concentration (20 μg/L) showed no significant difference compared to the control. GST activity (Figure 3B) was elevated in all OFL single-exposure groups (*p* < 0.01) and medium/high SMX single-exposure groups (*p* < 0.01), while the combined exposure showed no significant difference (*p* > 0.05). CAT activity (Figure 3C) was significantly increased only in the medium OFL single-exposure group (*p* < 0.01), with no significant changes in other groups (*p* > 0.05). MDA content (Figure 3D) was significantly elevated in all OFL and SMX single-exposure groups (*p* < 0.01), with no significant changes in the combined-exposure groups (*p* > 0.05).

Intestinal oxidative stress indicators showed a predominantly inhibitory trend. SOD activity (Figure 3E) was significantly reduced in all OFL single-exposure groups (*p* < 0.01), decreased in the low/medium SMX single-exposure groups (*p* < 0.01) but increased in the high SMX group (*p* < 0.01), and significantly downregulated in all combined-exposure groups (*p* < 0.01). GST activity (Figure 3F) decreased in the medium OFL single-exposure group (*p* < 0.05) and the low/medium SMX single-exposure groups (*p* < 0.01), and it plummeted by 22.9% in the medium combined-exposure group (*p* < 0.01). CAT activity (Figure 3G) showed no significant changes in all exposure groups (*p* > 0.05) except for a reduction in the high SMX single-exposure group. MDA content (Figure 3H) was significantly decreased in all single- and combined-exposure groups (*p* < 0.05), indicating that antibiotic exposure inhibited lipid peroxidation in intestinal tissue under the tested concentrations.

In summary, these two tissues exhibited distinct oxidative stress response patterns: the liver showed additive activation of antioxidant enzymes, while the intestine presented an inhibitory trend.

### 3.4. The Expression Changes in Antioxidant-Related Genes

**Figure 4:**
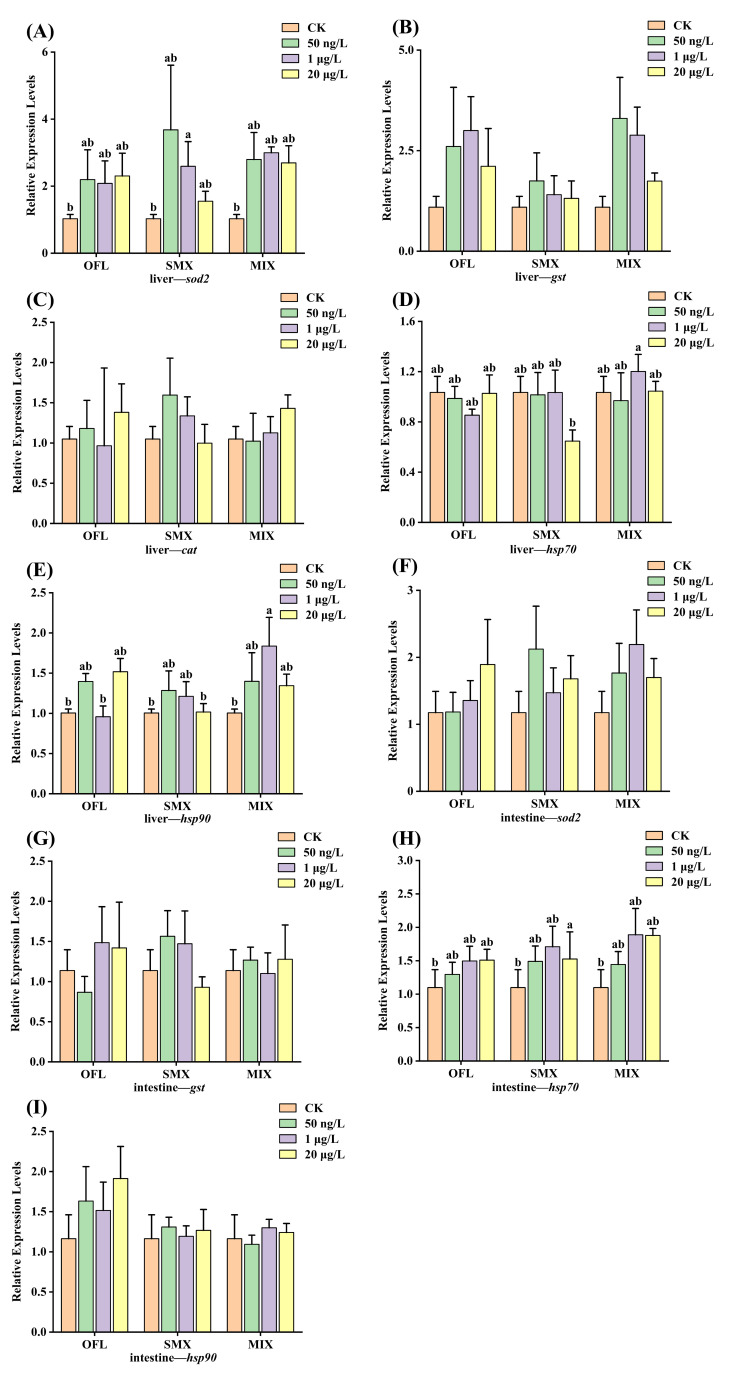
mRNA expression of antioxidant-related genes in the liver and intestine of mosquitofish exposed to ofloxacin and sulfamethoxazole alone and in combination. Liver—*sod2* (**A**); liver—*gst* (**B**); liver—*cat* (**C**); liver—*hsp70* (**D**); liver—*hsp90* (**E**); intestinal—*sod2* (**F**); intestinal—*gst* (**G**); intestinal—*hsp70* (**H**); intestinal—*hsp90* (**I**). Distinct lowercase letters (a, b) represent significant differences between groups (*p* < 0.05), and samples without letter labeling indicate no significant difference com-pared with the control group.

The mRNA expression levels of five antioxidant-related genes (*sod2*, *cat*, *gst*, *hsp70*, *hsp90*) in the liver and intestine exhibited tissue specificity. In the liver (Figure 4A–E), *sod2* gene expression was not significantly changed in all OFL single- and combined-exposure groups (*p* > 0.05), but it was significantly upregulated by 28.4% in the 1 μg/L SMX single-exposure group (*p* < 0.05). *The cat*, *gst* and *hsp70* genes displayed no significant fluctuations in all OFL/SMX single- and combined-exposure groups. *Hsp90* gene expression surged by 82.8% in the 1 μg/L combined-exposure group compared to the control (*p* < 0.01) and was significantly higher than the same-concentration OFL single-exposure group (*p* < 0.01).

In the intestinal tissue (Figure 4F–I), the *sod2*, *gst*, and *hsp90* genes showed no significant changes in all single- and combined-exposure groups. *Cat* gene expression was undetectable in partial samples, indicating low or absent expression in intestinal tissue. *hsp70* gene expression was significantly upregulated by 38.9% in the 20 μg/L SMX single-exposure group (*p* < 0.05), with no significant changes in the other groups (*p* > 0.05).

### 3.5. Tissue-Specific Toxic Response Biomarkers

**Figure 5:**
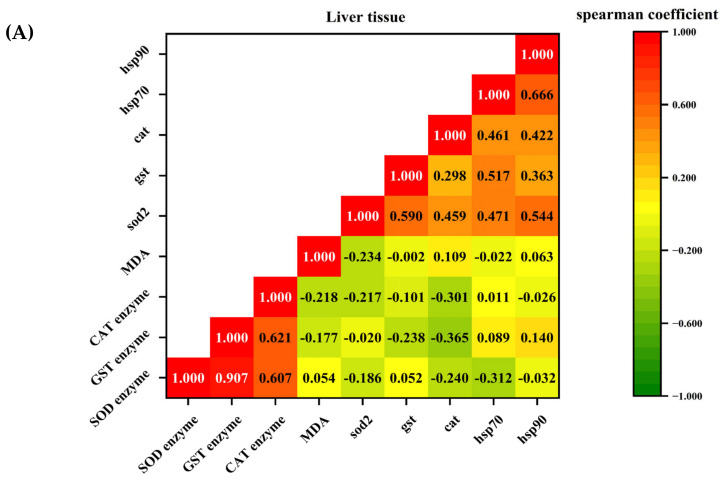

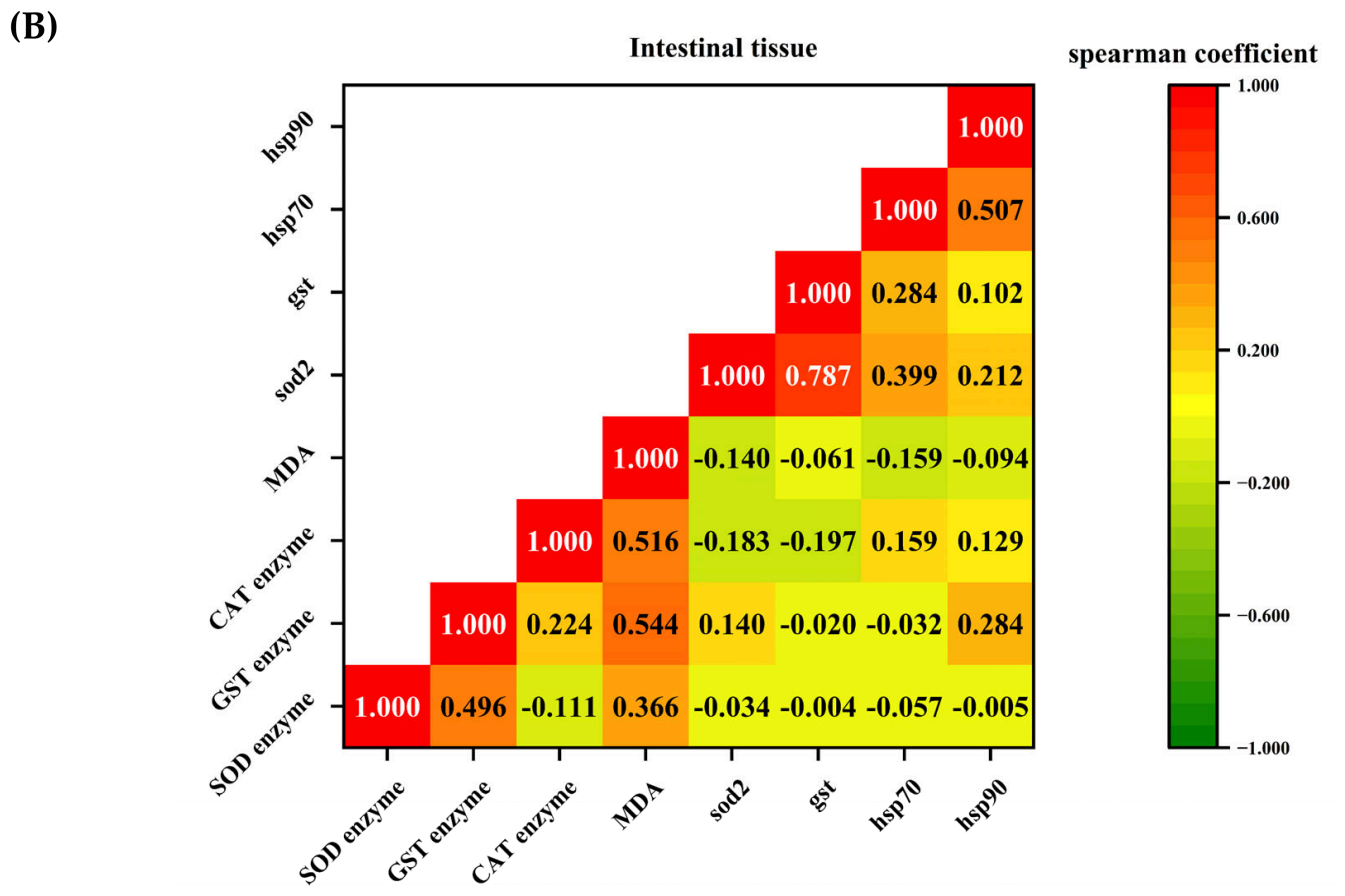
Spearman correlation heatmaps of oxidative stress and heat shock protein indices in the liver (**A**) and intestine (**B**) of mosquitofish following 30-day single and combined exposures to OFL and SMX.

Spearman correlation analysis revealed distinct tissue-specific coupling patterns of oxidative stress and heat shock responses in the mosquitofish under antibiotic exposure (Figure 5). In the liver, strong positive correlations were observed among antioxidant enzymes (SOD enzyme, CAT enzyme, GST enzyme; r = 0.607–0.907) and between heat shock protein genes (*hsp70* and *hsp90*, r = 0.666). Moderate positive correlations were detected between antioxidant genes and heat shock genes (*gst* and *sod2*, r = 0.590; *gst* and *hsp70*, r = 0.517), indicating high internal coordination within the antioxidant defense and heat shock systems, as well as moderate inter-system coupling. Malondialdehyde (MDA) showed weak negative correlations with all antioxidant indices, suggesting uncoupling between lipid peroxidation and antioxidant activation.

In the intestine, the overall correlation intensity was notably lower than that in the liver. The coordination among antioxidant enzymes was weakened (SOD-GST, r = 0.496; MDA-GST, r = 0.544; MDA-CAT, r = 0.516), and the positive correlation between *hsp70* and *hsp90* was reduced (r = 0.507), with limited inter-system coupling between antioxidant and heat shock responses. The strongest correlation was found between *sod2* and *gst* (r = 0.787), highlighting a core regulatory module of intestinal antioxidant defense.

### 3.6. Sequencing Quality of Intestinal Microbiota

High-throughput 16S rRNA sequencing of intestinal contents from the control and 20 μg/L OFL/SMX single- and combined-exposure groups generated high-quality sequencing data (Table S5). A total of 1,188,192 raw tags were obtained, and 1,016,040 valid taxon tags were retained after quality control and filtering, with the effective rate exceeding 85%. The number of singleton tags in each sample ranged from 8405 to 23,138, and the number of OTUs (operational taxonomic units) varied from 154 to 532. Specifically, the control group had an average of 99,487 sequences and 177 OTUs, the OFL exposure group had 100,512 sequences and 496 OTUs, the SMX exposure group had 94,628 sequences and 273 OTUs, and the combined OFL and SMX exposure group had 101,437 sequences and 194 OTUs.

### 3.7. Intestinal Microbiota Diversity, Community Structure and Functions

#### 3.7.1. Taxonomic Composition of Intestinal Microbiota

**Figure 6:**
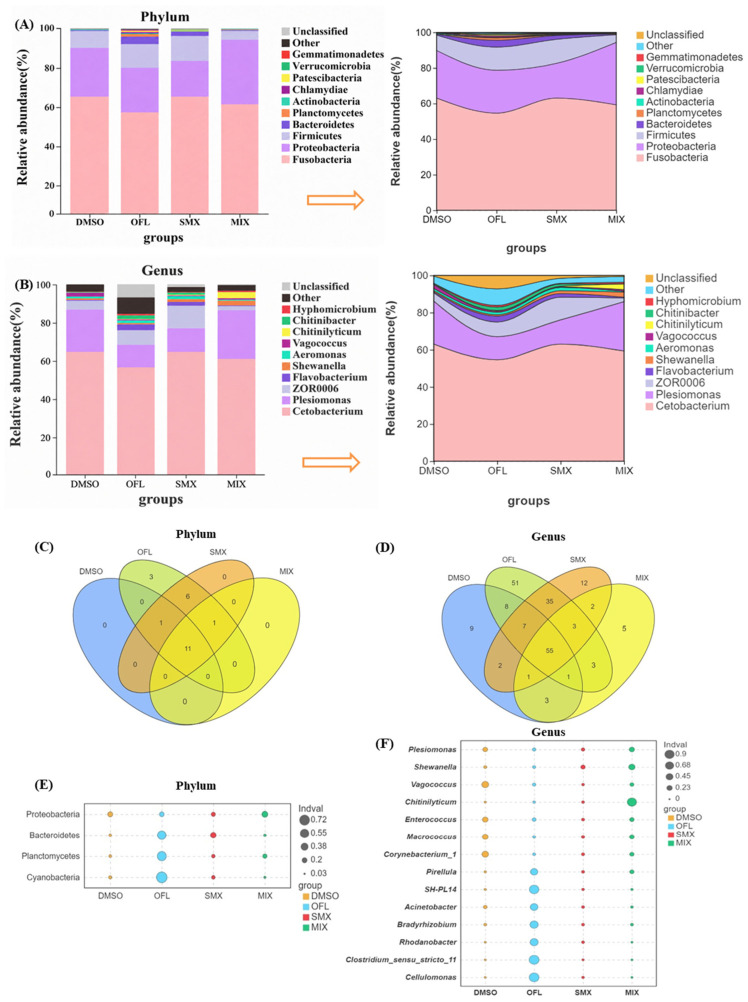
Taxonomic composition of gut microbiota in mosquitofish at the phylum (**A**) and genus (**B**) levels; Venn diagram illustrating the unique and shared bacterial phyla (**C**) and genera (**D**) across different treatment groups; bubble plot of indicator bacterial phyla (**E**) and genera (**F**) associated with different treatment groups.

At the phylum level (Figure 6A), Fusobacteria, Proteobacteria and Firmicutes were the dominant phyla across all groups, collectively accounting for >95% of the total microbial abundance. The composition of dominant phyla remained unchanged across all exposure groups. Compared with the DMSO control, the OFL group showed a marked increase in Bacteroidetes abundance (*p* < 0.05). The SMX group exhibited a similar community structure to the control. The MIX group displayed increased Proteobacteria and decreased Firmicutes (*p* < 0.05).

At the genus level (Figure 6B), *Cetobacterium* and *Plesiomonas* were the core dominant genera across all groups. Compared with the DMSO control, the OFL group showed a decrease in *Cetobacterium* and *Plesiomonas* and an elevated proportion of ZOR0006, “unclassified” and “Other” taxa, indicating the strongest disturbance. The SMX group maintained a similar abundance of *Cetobacterium* to the control, with decreased *Plesiomonas* and increased ZOR0006, suggesting a milder perturbation. The MIX group displayed a comparable disturbance pattern to the control, with slightly increased *Plesiomonas* and decreased ZOR0006.

River plots further confirmed the dynamic shifts in microbial composition at both taxonomic levels, visually demonstrating that OFL exposure drove the most pronounced compositional changes, while SMX exposure induced moderate alterations, and combined exposure (MIX) resulted in a community profile closer to the control group. These results indicate that OFL alone exerts a stronger impact on the gut microbial community than SMX, while combined exposure does not produce a synergistic or antagonistic effect.

#### 3.7.2. Indicator Taxa

At the phylum level (Figure 6C), 11 core phyla were shared across all groups. OFL harbored three unique phyla, while the control, SMX, and MIX had none. At the genus level (Figure 6D), 55 core genera were shared; OFL had the most unique genera (51), followed by SMX (12), the control (9) and MIX (5). Pairwise overlap was highest between OFL and SMX (35 genera).

Phylum-level indicator analysis (Figure 6E) showed that Cyanobacteria, Planctomycetes and Bacteroidetes were strongly associated with OFL (high indval). At the genus level (Figure 6F), the OFL group was characterized by multiple indicator genera (e.g., *Cellulomonas*, *Acinetobacter*), the control group by *Vagococcus* and *Corynebacterium_1*, and MIX group by *Chitinilyticum*, and the SMX group lacked highly specific indicators.

Overall, no exclusive phyla were identified in the combined-exposure group, whereas five exclusive genera were detected, among which *Chitinilyticum* served as the pivotal microbial biomarker. Such distinct alterations in gut microbial community structure demonstrated that combined exposure to OFL and SMX triggered interactive compensatory regulatory responses, revealing that their joint toxic effects cannot be simply categorized as synergistic or antagonistic action.

#### 3.7.3. Alpha Diversity

Alpha diversity indices (Chao1, ACE, Shannon, Simpson) and Goods coverage of intestinal microbiota are presented in Table S6. The Goods coverage of all samples was above 0.998, indicating that the sequencing depth fully covered the microbial community in the intestinal contents, and the results were representative. The OFL single-exposure group showed a significant increase in the Chao1 (504.0981, *p* < 0.05), ACE (522.7825, *p* < 0.05), and Shannon (2.9553, *p* < 0.05) indices compared to the control and mix group (Figure S1), indicating that OFL single exposure significantly increased the species richness and diversity of intestinal microbiota.

#### 3.7.4. Beta Diversity

**Figure 7:**
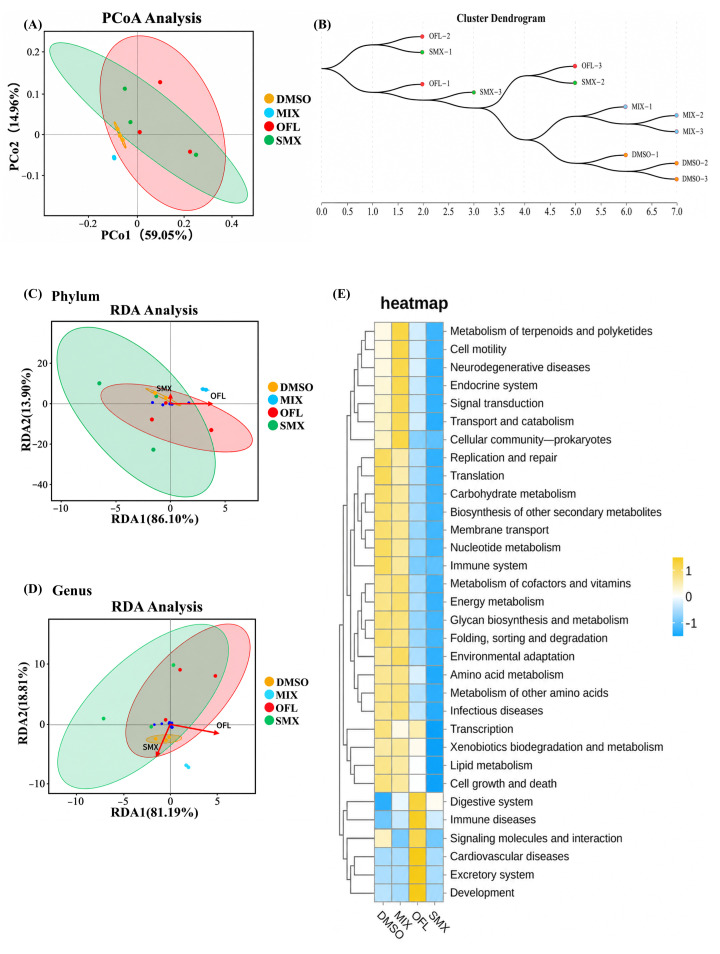
Principal coordinate analysis (PCoA) (**A**) of gut microbial communities among different groups; UPGMA clustering (**B**) of gut microbial communities based on Bray–Curtis distance; redundancy analysis (RDA) of microbial communities at the phylum (**C**) and genus (**D**) levels in response to different antibiotic treatments; PICRUSt-based functional prediction heatmap (**E**) of mosquitofish gut microbiota after OFL, SMX, and their mixture (MIX) exposure.

Principal Coordinates Analysis (PCoA) revealed clear separation of microbial communities across different groups (Figure 7A). The first two principal coordinates (PCo1 and PCo2) accounted for 59.05% and 14.96% of the total variation, respectively. The DMSO control group formed a tight and distinct cluster, while the MIX sample was positioned close to the control, indicating a minimal community shift. In contrast, the OFL and SMX groups showed extensive overlap along PCo1, suggesting highly similar antibiotic-induced microbiota perturbations.

Consistent with the PCoA results, UPGMA clustering (Figure 7B) confirmed that samples from the MIX and control groups clustered together and formed independent clades, while the OFL and SMX samples were more closely clustered, reflecting their phylogenetic proximity.

These results collectively demonstrate that single antibiotics (OFL, SMX) drive convergent community changes in the gut microbiota, whereas the combined treatment elicits a distinct, milder effect relative to controls.

#### 3.7.5. Contribution Analysis of Antibiotics

At the phylum level (Figure 7C), RDA1 and RDA2 explained 86.10% and 13.90% of the total variation, respectively. At the genus level (Figure 7D), the corresponding values were 81.19% and 18.81%, respectively. The DMSO control group formed a tight cluster near the origin, indicating minimal perturbation. The OFL and SMX groups were separated along RDA1 in opposite directions, reflecting distinct selection pressures. The OFL + SMX group occupied an intermediate position closer to the OFL group.

#### 3.7.6. Microbial Community Functional Pathway Analysis

Hierarchical clustering of KEGG pathways revealed two functional modules. Module 1 (basic metabolism and environmental adaptation, e.g., cell motility, signal transduction) was upregulated in the MIX group but downregulated in the SMX group. Module 2 (systemic and disease-related pathways, e.g., digestive system, immune diseases) was upregulated in the OFL group.

OFL specifically activated systemic pathways and suppressed basic metabolic pathways, while SMX strongly inhibited environmental response and cell interaction pathways. In contrast, the MIX group exhibited a compensatory regulatory pattern: it reversed the SMX-induced suppression of environmental adaptation pathways while avoiding the OFL-induced overactivation of systemic function pathways, indicating that the combined treatment counteracted OFL/SMX-induced functional activation/inhibition, resulting in a unique functional landscape distinct from single antibiotic treatments.

## 4. Discussion

### 4.1. Liver Injury and Oxidative Stress Responses

The liver is the primary detoxification and metabolic organ in fish, and hepatic histopathology is a well-established and sensitive biomarker for assessing the toxicity of aquatic contaminants [30,31,32]. Antibiotics are known to induce histological lesions, metabolic disturbances, and even organ damage in fish [33,34]. In this study, single exposure to OFL and SMX both induced cytoplasmic vacuolation, nuclear migration, cytolysis, pyknosis, and disordered cellular arrangement. Hepatic damage caused by OFL worsened in a concentration-dependent manner. Combined exposure further aggravated hepatic pathological lesions, with particularly pronounced adverse alterations observed at 20 μg/L, indicating that the mixture of the two antibiotics appears to act in an additive manner to exacerbate hepatic histopathological damage. These structural abnormalities are likely attributable to antibiotic-induced metabolic disorders, including inhibited protein synthesis, energy depletion, microtubule disruption, and altered substrate utilization [35].

Oxidative stress is a central mechanism underlying antibiotic-induced liver injury [2]. Antibiotic exposure disturbs hepatic energy metabolism by inhibiting aerobic glycolysis, promoting gluconeogenesis, and suppressing lipogenesis and fatty acid β-oxidation [36], thereby triggering endogenous ROS overproduction and redox imbalance [37,38]. Excessive ROS accumulation impairs the antioxidant defense system [39], induces lipid peroxidation, and ultimately leads to hepatic steatosis and vacuolar necrosis [40]. Among antioxidative biomarkers, SOD constitutes the first line of defense against superoxide radicals, GST is involved in glutathione-dependent phase II detoxification, and elevated MDA levels generally reflect enhanced membrane lipid peroxidation and loss of cell membrane integrity [41,42].

In the present study, single OFL and SMX exposures significantly increased SOD, GST, and MDA levels in the liver, indicating a compensatory activation of antioxidant and detoxification systems to counteract ROS-induced damage. However, these responses were insufficient to prevent structural injury. Consistent with previous reports, combined exposure to OFL and SMX enhanced toxic risks [43,44]. Notably, the high-dose combined group showed no further increase in enzyme activities but more severe histological damage, indicating that the hepatic antioxidant defense system had exceeded its compensatory threshold.

At the transcriptional level, only hepatic *sod2* (1 μg/L SMX group) and *hsp90* (1 μg/L combined group) were significantly upregulated, while other antioxidant genes remained stable. This weak transcriptional response reflects the temporal decoupling between mRNA expression and enzyme activity under chronic exposure (30 d), with oxidative stress responses shifting from rapid transcriptional regulation to long-term homeostasis remodeling [45,46]. This phenomenon is consistent with previous findings that antioxidant genes such as *sod*, *gst*, and *cat* in mussels were upregulated in the initial stage of SMX exposure but decreased or remained unchanged with prolonged exposure [47]. Alternatively, the weak transcriptional response may be attributed to the fact that protein-level enzyme activity is regulated by multiple genes, signaling pathways, and post-transcriptional factors, including small RNAs [48]. Under individual and combined OFL and SMX stress, the antioxidant defense system can counteract most oxidative damage, and the individual variations in antioxidant gene expression may represent fluctuations within a defensive buffering range to maintain homeostasis and prevent oxidative injury.

HSP90 is known to participate in the folding and stabilization of damaged proteins and to suppress apoptosis through cell survival-related pathways, thereby serving as an important compensatory mechanism for maintaining hepatocyte viability under pollutant-induced stress [49,50]. The specific upregulation of *hsp90* under the intermediate-dose combined exposure indicates severe protein damage and activated proteostasis repair, which helps stabilize misfolded proteins and inhibit apoptosis. Nevertheless, this compensatory response was inadequate to reverse lipid peroxidation and histological deterioration, resulting in overt liver injury.

Compared with single exposures, combined exposure induced attenuated SOD activity changes in both the liver and intestine, indicating reduced antioxidant responses at the enzymatic level. Nevertheless, this partial response failed to mitigate hepatic histopathological damage, confirming that additive toxic effects predominated at the tissue level. Similar toxicological manifestations were reported in previous studies; for instance, combined exposure to SMX and caffeine induced a weaker increase in SOD activity compared with single exposure [51]. CAT activity showed no significant changes across groups, which is consistent with observations in erythrocytes exposed to low concentrations of ciprofloxacin and enrofloxacin [52]. This stability may be attributed to the low sensitivity of CAT to environmentally relevant antibiotic concentrations, compensatory regulation by SOD and GST, or rapid recovery of CAT activity following transient induction under low-level stress. Collectively, 30 d exposure to OFL, SMX, and their mixture caused pronounced liver damage in mosquitofish, mediated by ROS-driven oxidative stress, disrupted antioxidant defense, and overwhelmed compensatory mechanisms.

### 4.2. Intestinal Injury and Oxidative Stress Responses

All exposure groups in this study exhibited intestinal structural damage, including muscular layer thickening, epithelial vacuolation and villus rupture. Similar intestinal lesions have been reported in tilapia exposed to 260 ng/L SMX and 420 ng/L oxytetracycline [17]. Intestinal mucosal integrity is essential for nutrient utilization and normal growth [53,54]; thus, the thickened muscular layer observed here likely represents an adaptive response to enhance nutrient absorption and compensate for antibiotic-induced metabolic inhibition [17,55,56].

Antibiotic exposure can induce endoplasmic reticulum stress in goblet cells and compromise the intestinal mucus barrier, independent of microbiota changes, thereby increasing bacterial invasion and intestinal inflammation [57]. Chronic exposure (six weeks) to low-dose antibiotics (260 ng/L SMX and 420 ng/L oxytetracycline) has also been shown to reduce intestinal goblet cell counts, suppress phosphatase activities, and trigger inflammatory responses in zebrafish [3]. In the present study, villus rupture destroyed the intestinal physical barrier, elevated permeability, and potentially promoted microbial and toxin translocation, leading to systemic toxicity. Notably, combined exposure did not cause synergistic or additive intestinal damage, likely due to low antibiotic bioaccumulation and effective mucosal compensatory repair capacity.

As the primary site of antibiotic absorption, the intestine is highly sensitive to antibiotics and can exhibit toxic responses in fish even at nanogram-per-liter levels [3]. Congruously, in this study, intestinal oxidative stress responses were distinct from those in the liver and were highly sensitive to nanogram-level single exposure. SOD and GST activities were significantly inhibited, while MDA levels decreased across most groups. Similar suppression of MDA has been reported in plants exposed to OFL [58]. A reduced MDA content does not indicate alleviated oxidative damage but rather accelerated clearance or altered metabolism of lipid peroxidation products [59]. Therefore, intestinal injury mainly resulted from suppressed antioxidant and detoxification capacity rather than lipid peroxidation.

Intestinal morphological damage was also mediated indirectly by antibiotic-induced gut microbiota dysbiosis. In fish, the intestinal microbiota regulates epithelial cell differentiation and proliferation, nutrient metabolism, lipid and cholesterol metabolism, and immune homeostasis; thus, microbial disturbance directly impairs mucosal structure and gut homeostasis [60]. In this study, OFL and SMX exposure restructured the core microbiota, reduced dominant symbionts, and expanded low-abundance taxa. Antibiotic-induced microbial dysbiosis has been linked to impaired mucosal gene expression, suppressed immune pathways, and triggered intestinal apoptosis and injury in grass carp [61]. Microbial shifts also disrupt energy and metabolic metabolism, further destabilizing intestinal homeostasis. For instance, in zebrafish, microbial dysbiosis is closely linked to disrupted energy metabolism and intestinal homeostasis [62]. In Atlantic salmon, florfenicol impairs gut microbiota and short-chain fatty acid synthesis, further disturbing glucose metabolism [63]. Thus, in the present study, intestinal damage in the mosquitofish arose from both direct antibiotic toxicity and indirect microbial dysregulation.

At the transcriptional level, antioxidant gene expression was relatively stable, consistent with reports of uncoupled mRNA and enzyme activities under long-term environmental stress [64]. Only *hsp70* was significantly upregulated under high-dose SMX, indicating a mild protective response. Collectively, the intestine exhibited tissue-specific sensitivity to OFL and SMX, with injury driven by barrier disruption, antioxidant suppression, and microbiota dysbiosis rather than marked transcriptional activation.

### 4.3. Intestinal Microbiota Diversity and Community Alterations

Gut microbiota is essential for maintaining host physiological homeostasis and immune regulation [65]. In this study, environmentally relevant concentrations of OFL and SMX did not alter the dominant gut microbial composition at the phylum level, with Fusobacteria, Proteobacteria, and Firmicutes still accounting for over 95% of the total community. This stability may be attributed to the balance between antibiotic bioaccumulation and biotransformation in fish tissues [47]. These dominant phyla were consistent with those reported in grass carp and crucian carp, which are also dominated by Firmicutes, Bacteroidetes, Spirochaetes, and Proteobacteria [66,67]. Such a conserved microbial community structure plays key roles in intestinal nutrient absorption, digestion, and immune function [68].

An elevated Firmicutes/Bacteroidetes (F/B) ratio is typically considered a key feature of gut microbial dysbiosis and metabolic disorders [69,70]. In the present study, however, the average F/B ratio was markedly decreased in all treatment groups compared with the control (17.2), with ratios of 3.3, 5.5, and 10.3 in the OFL, SMX, and combined-exposure groups, respectively. This decrease does not necessarily indicate a healthy state, as an excessive reduction in the F/B ratio may instead reflect gut ecological disruption, with the effect ranking as OFL alone > SMX alone > combined exposure.

A previous report showed that OFL alone increased the Shannon diversity index in *Phragmites australis* rhizosphere microbes, while an OFL + SMX mixture showed no significant difference from the control [58]. Consistently, alpha diversity analysis revealed that single OFL exposure significantly increased microbial richness and diversity in the mosquitofish, whereas combined exposure showed no significant difference compared with the control. Similar increases in gut microbial diversity under low-dose antibiotic stress have been reported in zebrafish [71] and mice [72]. This phenomenon occurs because low concentrations of antibiotics inhibit dominant taxa and create ecological niches for rare bacteria, even though high doses of these antibiotics are typically used to suppress bacterial growth. Beta diversity results further confirmed that single OFL and SMX exposures reshaped microbial community structure, while the combined-exposure group remained highly similar to the control.

At the genus level, *Cetobacterium*, *Plesiomonas*, and unnamed genus ZOR0006 were dominant. *Cetobacterium* is critical for vitamin B12 supply and intestinal barrier stability [73], while some *Plesiomonas* species are opportunistic pathogens [74]. Single OFL exposure significantly reduced the abundance of *Cetobacterium* and *Plesiomonas* while increasing the proportion of rare taxa. This pattern indicates the disruption of core symbiotic microbiota and reduced microecological stability. In contrast, the combined exposure only induced mild shifts in the gut microbiota, which points to the presence of interactive compensatory mechanisms in the fish. Notably, previous studies have shown that Proteobacteria is one of the dominant core phyla in the zebrafish gut [75], and the abundance of *Cetobacterium* changed markedly in mosquitofish after rifampicin exposure [76]. These findings indicate that different classes of antibiotics may exert distinct selective pressures on the core gut microbiota.

PCoA, UPGMA and RDA analyses consistently revealed that single exposure to OFL and SMX triggered similar shifts in the microbial community, whereas the combined treatment clustered closely with the control group. The combined exposure did not induce any unique phyla and only a few unique genera, with *Chitinilyticum* serving as the characteristic biomarker. Functionally, OFL activated systemic and disease-related pathways, while SMX suppressed pathways associated with environmental adaptation and cellular interaction. In contrast, the combined exposure exerted a compensatory regulatory effect, reversing functional inhibition and avoiding excessive pathway activation. Similar studies have demonstrated that chronic antibiotic exposure can cause gut microbiota dysbiosis in zebrafish, further influencing the intestinal mucosal barrier, goblet cell abundance, and host stress- and immune-related processes [69,77].

In summary, single OFL and SMX exposures disrupted gut microbial structure and function, with OFL exerting more pronounced toxic effects. Under combined exposure, no synergistic or additive disturbance effect was observed in the fish. Antibiotics may affect mosquitofish health not only through direct toxicity but also by reshaping gut microbiota and interfering with metabolic homeostasis.

### 4.4. Comparison Between Single and Combined Exposures

Single and combined exposures to OFL and SMX caused tissue-specific interactive effects in mosquitofish, which were determined by differential antibiotic accumulation, metabolic capacity, and antioxidant reserves among tissues [78].

In the liver, combined exposure at the concentration of 20 μg/L induced additive histopathological damages, manifested as markedly aggravated cytolysis and cytoplasmic vacuolar degeneration. As the primary organ responsible for detoxification, metabolism, and immune regulation [79], the liver exhibits high antibiotic bioaccumulation, as widely reported in previous studies [80,81,82,83,84]. Single exposure activated SOD and GST as a compensatory response, but high-dose combined exposure exceeded the defense threshold, leading to severe injury even with no further increase in enzyme activities.

As the primary absorption site, the intestine is highly sensitive to antibiotics but has lower bioaccumulation and stronger mucosal repair capacity. Antioxidant responses were characterized by inhibited SOD and GST and decreased MDA, which did not further deteriorate under combined exposure.

In gut microbiota, combined exposure showed non-additive compensatory effects. Unlike the strong disturbance caused by single OFL exposure, combined exposure maintained community structure and function close to the control, owing to complementary antibacterial spectra and mutual restriction of the two antibiotics.

Overall, OFL showed higher toxicity than SMX across all tissues and microbial indicators. Combined exposure did not merely amplify the toxic effects of individual antibiotics but also exerted tissue-dependent interactive effects: additive toxicity was observed in the liver, whereas interactive compensatory regulatory mechanisms were activated in the intestine and intestinal microbiota. This tissue-specific pattern highlights the necessity of evaluating combined toxicity in multi-organ and multi-index systems for ecological risk assessment of antibiotic mixtures.

## 5. Conclusions

Environmentally relevant concentrations of OFL and SMX induced clear tissue-specific toxicity in mosquitofish after 30 days of exposure. The liver was more sensitive than the intestine, showing stronger oxidative stress responses and more severe histopathological damage. OFL generally exerted greater toxicity than SMX under single exposure for both tissue and microbial endpoints, whereas co-exposure further aggravated hepatic injury, especially in the 20 μg/L groups. In the intestine, toxicity was mainly reflected by barrier disruption and suppression of antioxidant and detoxification defenses. In addition, antibiotic exposure altered the gut microbial community by reshaping the relative abundance of core taxa and predicted microbial functions. Overall, OFL and SMX may threaten aquatic organism health through both direct tissue damage and indirect disruption of intestinal microecological homeostasis.

## Data Availability

The original contributions presented in this study are included in this article/the Supplementary Materials. Further inquiries can be directed to the corresponding author.

## Supplementary Materials

The following supporting information can be downloaded at https://www.mdpi.com/article/10.3390/toxics14060457/s1: Table S1 qPCR primer sequence of target genes. Table S2 Nominal and actual measured concentrations of OFL and SMX in exposure solutions. Table S3 Hepatic histopathological alterations in adult mosquitofish after 30-day single and combined exposure to OFL and SMX. Table S4 Intestinal histopathological alterations in adult mosquitofish after 30-day single and combined exposure to OFL and SMX. Table S5 Summary of high-throughput sequencing data processing for intestinal microflora of *Gambusia affinis*. Table S6 Alpha diversity analysis of intestinal microbiota in mosquitofish. Figure S1 Alpha diversity indices (Chao1, ACE, Shannon) of intestinal microbiota in mosquitofish from different groups. Stars (*) indicate significant different from the control and mix group (*: *p* < 0.05; **: *p* < 0.01).
