# Supplementary material for: Combined Toxicity of Ofloxacin and Sulfamethoxazole at Environmentally Relevant Concentrations in Mosquitofish: Histopathological Damage, Oxidative Stress, and Gut Microbiota Alterations
Source: Toxics. 2026 May 23;14(6):457. doi: 10.3390/toxics14060457 (PMC13306796; doi:10.3390/toxics14060457)
Supplement: Supplementary file 1 [file toxics-14-00457-s001.zip › toxics-4268942-supplementary.pdf]

Combined Toxicity of Ofloxacin and Sulfamethoxazole at Environmentally Relevant Concentrations in Mosquitofish: Histopathological Damage, Oxidative Stress, and Gut Microbiota Alterations

Xu Ding [a], Xin Li [a], Haojie Liu [a], Zhong Li [b], Yangchun Xia [c], Yanpeng Liang [a b c], Honghu Zeng [a b c], Xiaohong Song [a b c *]

[a]Guangxi Key Laboratory of Environmental Pollution Control Theory and Technology, Guilin University of Technology, Guilin 541006, China

[b]University Engineering Research Center of Watershed Protection and Green Development, Guangxi, Guilin University of Technology, Guilin 541006, China

[c]3. Key Laboratory of Carbon Emission and Pollutant Collaborative Control, Education Department of Guangxi Zhuang Autonomous Region, Guilin University of Technology, Guilin, 541006, China

* Corresponding author, Xiaohong Song, Email: songxh@glut.edu.cn

College of Environmental Science and Engineering, Guilin University of Technology, No.12, Jiangan Road, Guilin, 541004, China

Table S1 qPCR primer sequence of target genes

Genes	Forward primer sequence (5'-3')	Reverse primer sequence (5'-3')
cat	CCATCTTCTTCATCAGGGACGC	GGGTTTGAGGGTTTCGCTTCT
gst	TGCTCGCCATCAATCCCAGG	AAGCACCGTAGGACTCGTTCAG
gapdh	TTACTTCAATGACTCCCAGCG	TCAGGGATGACCTTGCCAACAG
hsp70	TTACTTCAATGACTCCCAGCG	TTACTTCAATGACTCCCAGCG
hsp90	GAGGACCTGCCCCTAAACA	GATTCTGGGAGTCTTCGTGG
sod2	TACTGGGCACTGACTAAGGAGC	GGCATTGATGTATGGGAGCA

Table S2 Nominal and actual measured concentrations of OFL and SMX in exposure solutions

Medicine	group	Nominal concentration	Actual detected concentration
OFL	OFL-L	50 ng/L	47.230±4.584 ng/L
	OFL-M	1 μg/L	1.104±0.0320 μg/L
	OFL-H	20 μg/L	22.168±4.439 μg/L
	MIX-L	50 ng/L	47.089±17.650 ng/L
	MIX-M	1 μg/L	0.977±0.085 μg/L
	MIX-H	20 μg/L	23.382±2.118 μg/L
SMX	SMX-L	50 ng/L	36.701±8.418 ng/L
	SMX-M	1 μg/L	0.642±0.090 μg/L
	SMX-H	20 μg/L	18.129±2.128 μg/L
	MIX-L	50 ng/L	47.807±3.022 ng/L
	MIX-M	1 μg/L	0.562±0.112 μg/L
	MIX-H	20 μg/L	18.438±0.654 μg/L

Table S3 Hepatic histopathological alterations in adult mosquitofish after 30-day single and combined exposure to OFL and SMX

Group	Cytoplasmic vacuolation	Cytolysis	Condensed nuclei	Nuclear fragmentation
DMSO	−	−	−	−
OFL-50 ng/L	++	++	++	+
OFL-1 μg/L	+++	++	+++	+
OFL-20 μg/L	+++	++	+++	++
SMX-50 ng/L	+++	++	++	+
SMX-1 μg/L	++	++	++	++
SMX-20 μg/L	+	++	+++	+
MIX-50 ng/L	++	++	+++	++
MIX-1 μg/L	++	++	+++	++
MIX-20 μg/L	+++	+++	++	+++

Note: Negative / none or occasional (−), mild (+), moderate (++), severe (+++).

Table S4 Intestinal histopathological alterations in adult mosquitofish after 30-day single and combined exposure to OFL and SMX

Group	muscular layer thickening	vacuolar degeneration	villus rupture
DMSO	−	−	−
OFL-50 ng/L	+	++	++
OFL-1 μg/L	++	++	++
OFL-20 μg/L	++	++	++
SMX-50 ng/L	+	+	++
SMX-1 μg/L	+	++	+++
SMX-20 μg/L	++	++	++
MIX-50 ng/L	+	+	++
MIX-1 μg/L	+	++	+++
MIX-20 μg/L	+	+++	+++

Note: Negative / none or occasional (−), mild (+), moderate (++), severe (+++).

Table S5 Summary of high-throughput sequencing data processing for intestinal microflora of *Gambusia affinis*

Group	Sample	Total Tags	Taxon Tags	Singleton Tags	OTUs
Control	DMSO-1	87910	78827	9083	178
	DMSO-2	105216	96811	8405	178
	DMSO-3	105334	96107	9227	175
OFL	OFL-H-1	108726	94071	14655	441
	OFL-H-2	101395	86547	14848	532
	OFL-H-3	91416	70791	20625	514
SMX	SMX-H-1	87691	64553	23138	494
	SMX-H-2	94389	79871	14518	172
	SMX-H-3	101804	88485	13319	154
MIX	MIX-H-1	94529	81017	13512	188
	MIX-H-2	103301	88277	15024	191
	MIX-H-3	106481	90683	15798	203

Table S6 Alpha diversity analysis of intestinal microbiota in mosquitofish

Group	Sample	Chao1	ACE	shannon	simpson	Goods coverage
Control	DMSO-1	210.5000	207.7837	1.7308	0.5193	0.9995
	DMSO-2	201.3750	205.0226	1.8628	0.5503	0.9996
	DMSO-3	197.5455	198.2643	1.9416	0.5680	0.9997
	Average	203.1402	203.6902	1.8451	0.5459	0.9996
OFL	OFL-H-1	444.2292	455.3071	2.9572	0.6491	0.9997
	OFL-H-2	539.7044	559.7477	3.5232	0.7600	0.9994
	OFL-H-3	528.3607	553.2927	2.3855	0.5952	0.9990
	Average	**504.0981***	**522.7825***	**2.9553***	0.6681	0.9994
SMX	SMX-H-1	515.4570	540.5920	2.8150	0.7000	0.9987
	SMX-H-2	185.2857	194.9246	1.8785	0.5456	0.9996
	SMX-H-3	201.1429	192.1913	1.3192	0.3878	0.9995
	Average	300.6285	309.2360	2.0042	0.5445	0.9993
MIX	MIX-H-1	207.5238	225.0815	1.9406	0.5728	0.9995
	MIX-H-2	198.4595	205.0582	1.9570	0.5777	0.9997
	MIX-H-3	226.5227	244.7345	1.9434	0.5775	0.9995
	Average	210.8353	224.9581	1.9470	0.5760	0.9996

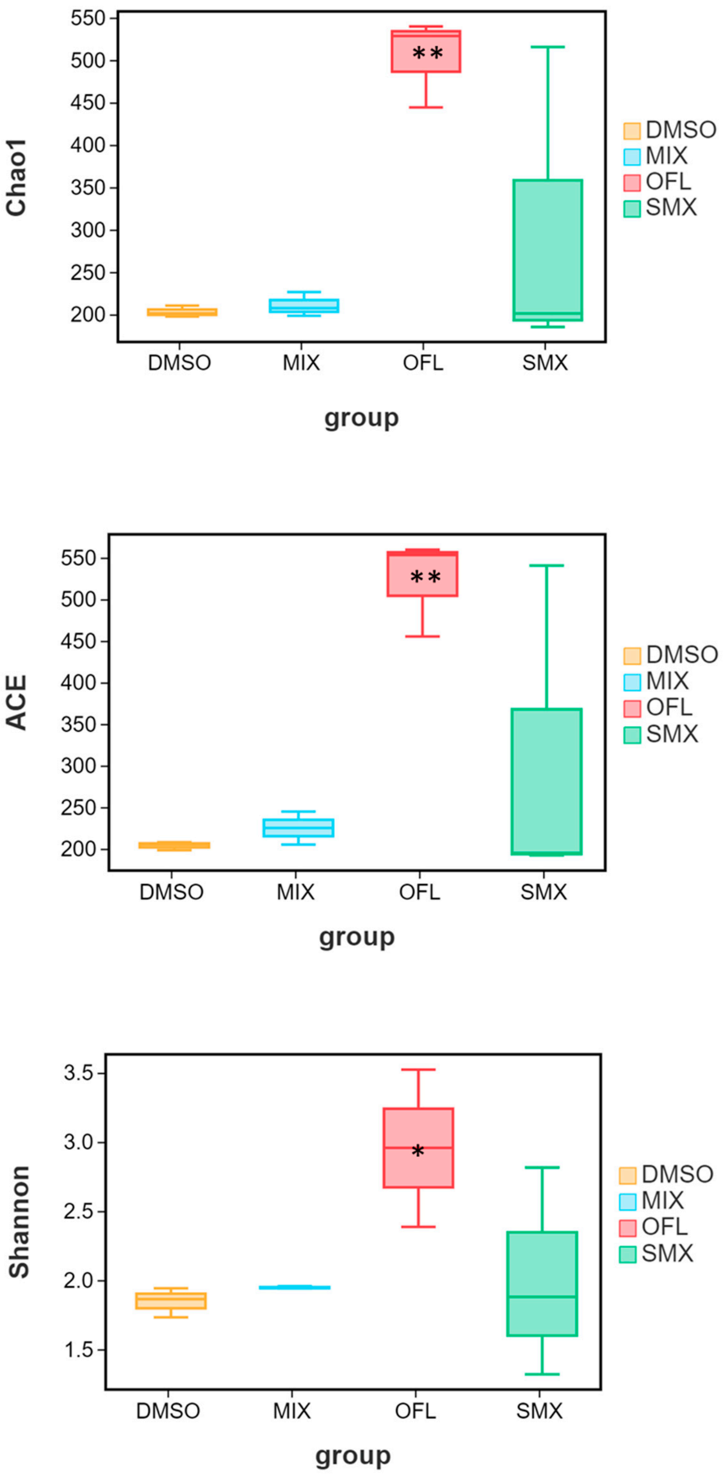

Figure S1 Alpha diversity indices (Chao1, ACE, Shannon) of intestinal microbiota in mosquitofish from different groups. Stars (*) indicate significant different from the control and mix group (*: $P<0.05$; **: $P<0.01$).
